# Cardioprotective Effect of (*Z*)-2-Acetoxy-3-(3,4-Dihydroxyphenyl) Acrylic Acid: Inhibition of Apoptosis in Cardiomyocytes

**DOI:** 10.1155/2020/8584763

**Published:** 2020-04-29

**Authors:** Wei Guo, Zhijun Wang, Hao Jue, Chunnan Dong, Cheng Yang

**Affiliations:** ^1^Department of Pharmacology, School of Pharmacy, Fudan University, No. 826, Zhangheng Road, Pudong New District, Shanghai 201203, China; ^2^Department of Cardiac Surgery, Zhongshan Hospital, Fudan University, No. 180, Fenglin Road, Shanghai 200032, China

## Abstract

**Background:**

Although many studies have been performed to elucidate the molecular mechanisms of heart failure, an effective pharmacological therapy to protect cardiac tissues from severe loss of contractile function associated with heart failure after acute myocardial infarction (MI) has yet to be developed.

**Methods:**

We examined the cardioprotective effects of (*Z*)-2-acetoxy-3-(3,4-dihydroxyphenyl) acrylic acid, a new compound with potent antioxidant and antiapoptotic activities in a rat model of heart failure. (*Z*)-2-Acetoxy-3-(3,4-dihydroxyphenyl) acrylic acid was systemically delivered to rats 6 weeks after MI at different doses (15, 30, and 60 mg/kg). Cardiac function was assessed by hemodynamic measurements. The expression of proinflammatory cytokines, apoptosis-related molecules, and markers of adverse ventricular remodeling was measured using RT-PCR and Western blot.

**Results:**

Treatment with (*Z*)-2-acetoxy-3-(3,4-dihydroxyphenyl) acrylic acid significantly improved cardiac function, in particular by increasing *dP*/*dt*. Simultaneously, the expression of the proinflammatory cytokines TNF-*α* and IL-1*β* was markedly reduced in the treatment group compared with the MI group. In addition, (*Z*)-2-acetoxy-3-(3,4-dihydroxyphenyl) acrylic acid-treated tissues displayed decreased expression of Bax, caspase-3, and caspase-9 and increased expression of Bcl-2, which was in part due to the promotion of Akt phosphorylation.

**Conclusion:**

These data demonstrated that (*Z*)-2-acetoxy-3-(3,4-dihydroxyphenyl) acrylic acid possesses potent cardioprotective effects against cardiac injury in a rat model of heart failure, which is mediated, at least in part, by suppression of the inflammatory and cell apoptosis responses.

## 1. Introduction

A severe loss of contractile function is the main characteristic of end-stage heart failure. Pathological analyses of the myocardium from patients with end-stage heart failure indicate a loss of cardiomyocytes as well as fibrosis and cardiomyocyte hypertrophy [1]. The collective evidence now demonstrates that a defect in the myocardium is a main reason for heart failure. Therefore, recent studies have focused on identifying chemical agents that can prevent cardiac damage following MI. Caspase-9 appears to play an important role in cell apoptosis. Activation of caspase-9 can initiate a proteolysis cascade by cleaving other caspases, such as caspase-3, which are effectors that carry out the cellular death program [2, 3]. Meanwhile, protein kinase (Akt), a key effector in the antiapoptosis survival pathway, can inactivate glycogen synthase kinase 3*β* (GSK-3*β*) by phosphorylation, thereby blocking caspase-3 activation [4]. Additionally, an imbalance of proapoptotic versus prosurvival members in the Bcl-2 family plays a major role in the apoptosis of cardiomyocytes and heart failure [5, 6].

Furthermore, recent evidence from both animal and human studies suggests that increased production of inflammatory cytokines and cardiac cell oxidative stress are associated with a poor prognosis following MI and may play a critical role in the pathogenesis and progression of heart failure. Indeed, myocardial cell death is reported to trigger an acute inflammatory reaction that leads to chemokine and cytokine production and the recruitment of macrophages that results in leukocyte infiltration of the infract area [7–10]. In addition, it is also known that after myocardial ischemic injury, cytokines such as TNF-*α* and IL-6 are also released and that these molecules can promote cell death signaling cascades in cardiac tissues [11]. Taken together, these findings indicate that inhibiting cardiomyocyte apoptosis and reducing inflammatory responses in cardiac tissues may prevent the loss of the myocardium and thereby reduce the adverse effects associated with heart failure.

In the present study, we assessed the therapeutic effects of (*Z*)-2-acetoxy-3-(3,4-dihydroxyphenyl) acrylic acid in a rat model of heart failure. (*Z*)-2-Acetoxy-3-(3,4-dihydroxyphenyl) acrylic acid is a compound with two phenolic hydroxyl groups. It is reported that caffeic acid phenethyl ester, which also has two phenolic hydroxyl groups, has antiviral, anti-inflammatory, antioxidant, and immunomodulatory properties, in addition to being a potent and specific inhibitor of NF-*κ*B activation [12–17].

In one of our previous studies, we demonstrated that (*Z*)-2-acetoxy-3-(3,4-dihydroxyphenyl) acrylic acid mediates potent protective effects against hypoxia-induced cellular damage by blocking oxidative stress and apoptosis [18]. These findings encouraged us to investigate the hypothesis that administration of (*Z*)-2-acetoxy-3-(3,4-dihydroxyphenyl) acrylic acid could modulate local myocardial inflammation, inhibit cardiomyocyte apoptosis, attenuate adverse ventricular remodeling, and subsequently improve cardiac performance in post-MI chronic heart failure.

## 2. Methods

### 2.1. Experimental Heart Failure and Administration of (*Z*)-2-Acetoxy-3-(3,4-Dihydroxyphenyl) Acrylic Acid

The rats used in the current study were supplied by the experimental animal center of Fudan University. Adult male Sprague-Dawley rats weighing 230-250 g (8-10 w) were used for the study. The animals were housed with a 12 h light-12 h dark cycle and had free access to standard pellet food and water throughout the experiment. All of the experiments were approved by the local ethics committee of Fudan University.

For the experiments, the adult male Sprague-Dawley rats were randomly divided into five groups: a sham operation group, MI group, and MI with drug treatment groups (15, 30, and 60 mg/kg). The drug was dissolved in DMSO (dimethyl sulfoxide). The drug was injected intraperitoneally 6 weeks after surgery once daily. The sham operation group and the MI group were injected only with DMSO according to animal weight. Prior to the induction of MI, the animals were intubated and artificially ventilated with a rodent ventilator (DHX-150, China) under anesthesia with 7% chloral hydrate (60 mg/kg i.p.). The left anterior descending coronary artery was ligated with a 5-0 suture, 1-2 mm below the left atrial appendage [19]. Six weeks after surgery, after measuring the hemodynamic parameters, the hearts from rats representing each group were excised and washed with PBS. The whole LV was stored at -80°C.

### 2.2. Assessment of Cardiac Function Hemodynamic Measurements

Left ventricular pressures were measured via a saline-filled cannula that was inserted through the right carotid artery and connected to a pressure transducer. The cannula was inserted into the left ventricle to monitor left ventricular systolic pressure (LVSP) and left ventricular end-diastolic pressure (LVEDP), as well as to measure the maximum rate of the rise of the left ventricular pressure rising rate (*dP*/*dt*).

### 2.3. Enzymatic Activity Assays

Six weeks after surgery, the whole blood of the rats was centrifuged (3000 r/min, 10 min, 4°C) to obtain serum. The antioxidant enzyme activities of malondialdehyde (MDA), catalase (CAT), superoxide dismutase (SOD), glutathione (GSH), glutathione peroxidase (GSH-Px), and glutathione S-transferase (GST) were measured in the serum. MDA, CAT, SOD, GSH, GSH-Px, and GST levels were measured by using a commercially available kit according to the manufacturer's instructions (Jiancheng Institute of Biotechnology, Nanjing, China).

MDA activity was measured by adding 1 ml of the standard agent to the standard tubes. Next, 1 ml of absolute ethyl alcohol was added to the blank tubes. Then, 1 ml of the sample was added to the sample tubes and control tubes. The tubes were shaken for a long period. Then, 3 ml of reagent 2 was added to all the tubes, and then, 1 ml of reagent 3 was added to the standard tubes, blank tubes, and sample tubes. Next, 1 ml of 50% glacial acetic acid was added to the control tubes. The tubes were mixed and covered with plastic wrap. A small hole was made in the plastic wrap. The tubes were incubated at 95°C for 40 minutes and cooled in flow water. The tubes were centrifuged at 3000 to 4000 rpm for 10 minutes. The absorbance of the supernatant was measured using a plate reader at 532 nm.

CAT activity was measured by first preparing positive control tubes and sample tubes. Then, 0.1 ml of serum was added to the sample tubes, and 1.0 ml of reagent 1 and 0.1 ml of reagent 2, both kept at 37°C, were added to each tube. They were incubated on a shaker at 37°C for precisely 1 min. After that, 1.0 ml of reagent 3 and 0.1 ml of reagent 4 were added to all tubes. Then, 0.1 ml of the serum was added to the positive control tubes. Finally, the absorbance was read at 405 nm using a plate reader.

To measure GSH activity, the preparation of the sample was as follows: 0.5 ml of the sample and the buffer of reagent 1 were mixed and centrifuged at 3000 to 4000 rpm for 10 minutes. Then, 1 ml of the sample supernatant was added to sample tubes, while 1.0 ml of reagent 1 and 1.0 ml of standard 20 *μ*mol/l aqueous GSH were added to the blank tubes and the positive control tubes. Then, 1.25 ml of reagent 2, 0.25 ml of reagent 3, and 0.05 ml of reagent 4 were added to each tube. The absorbance was read at 420 nm using a plate reader.

SOD activity was measured as follows: 0.5 ml of the sample and the buffer of 1.0 ml reagent 1 were mixed; meanwhile, 0.5 ml of water and the buffer of 1.0 ml reagent 1 were mixed as the positive control. Then, 0.1 ml of reagents 2, 3, and 4 was added to the sample tubes and the positive control tube. Then, all of the tubes were incubated at 37°C for precisely 40 minutes. 2.0 ml of color reagents were added to each tube. Finally, all the tubes were placed at room temperature for 15 minutes. The absorbance was read at 550 nm using a plate reader.

GSH-Px activity was measured by first adding 0.2 ml of aqueous 1 mmol/l GSH to the control tubes and the sample tubes. Then, 0.1 ml of the sample was added to the sample tubes. All of the tubes and reagent 1 were kept at 37°C for 5 minutes. Then, 0.1 ml of reagent 1 was added to each tube. All of the tubes were incubated at 37°C for precisely 5 minutes. Two milliliters of reagent 2 were added to every tube, and 0.1 ml of the sample was added to the control tubes. The tubes were centrifuged at 3000 to 4000 rpm for 10 minutes. One milliliter of the supernatant from the control tubes and the sample tubes was transferred to new tubes labeled the same as the old tubes. Simultaneously, 1 ml of the GSH standard buffer was added to the blank tubes and 1 ml of the aqueous 20 *μ*mol/l GSH was added to the standard tubes. Then, 1 ml of reagent 3, 0.25 ml of reagent 4, and 0.05 ml of reagent 5 were added to all of the tubes. The plate was covered and incubated on a shaker for 15 minutes at room temperature. Finally, the absorbance was read at 412 nm using a plate reader.

GST activity was measured by first adding 0.3 ml of aqueous 1 mmol/l GSH to the control tubes and the sample tubes. Then, 0.1 ml of the sample was added to the sample tubes. The tubes were mixed and incubated at 37°C for 30 minutes. Then, 2 ml of reagent 2 was added to all the tubes. Next, 0.1 ml of the sample was added to the control tubes. The tubes were centrifuged at 3000 to 4000 rpm for 10 minutes. One milliliter of the supernatant from the control tubes and the sample tubes was transferred to new tubes labeled the same as the old tubes. Then, 2 ml of the GSH buffer and 2 ml of aqueous 20 *μ*mol/l GSH were added to the blank tubes and standard tubes. Finally, 2 ml of reagent 3 and 0.5 ml of reagent 4 were added to all the tubes. The tubes were incubated for 15 minutes at room temperature, and the absorbance was read at 412 nm using a plate reader.

### 2.4. Reverse Transcription Polymerase Chain Reaction (RT-PCR)

Total RNA was isolated from cardiac myocytes by using TRIzol (Invitrogen, Carlsbad, CA) as previously described. The RNA concentration was determined by measuring the absorbance at 260 nm. Reverse transcription (RT) was conducted by using a PrimeScript™ 1st Strand cDNA Synthesis Kit according to the manufacturer's recommended protocol. The primers for RT-PCR were synthesized by Shanghai Sangon Biological Engineering Technology and Service Co., Ltd, China. The primers used for amplification were synthesized as follows: TNF-*α* forward 5′-ATGAGCACGGAAAGCATGATCCGA-3′, reverse 5′-CCAAAGTAGACCTGCCCGGACTC-3′; IL-1*β* forward 5′-ATGGCAACTGTCCCTGAACTCAACT-3′, reverse 5′-CAGGACAGGTATAGATTCAACCCCTT-3′; Bcl-2 forward 5′-CGGGAGAACAGGGTATGA-3′, reverse 5′-CAGGCTGGAAGGAGAAGAT-3′; Bax forward 5′-GCAGGGAGGATGGCTGGGGAGA-3′, reverse 5′-TCCAGACAAGCAGCCGCTCACG-3′; caspase-3 forward 5′-CTGGACTGCGGTATTGAG-3′, reverse 5′-GGGTGCGGTAGAGTAAGC-3′; TGF-*β* forward 5′-CGCAACAACGCAATCTATG-3′, reverse 5′-AGCCCTGTATTCCGTCTCC-3′; collagen-1 forward 5′-CATAAAGGGTCATCGTGGCT-3′, reverse 5′-TGTTCTCAATCTGCTGGCTCA-3′; and GAPDH forward 5′-TTCAACGGCACAGTCAAGG-3′, reverse 5′-CGGCATGTCAGATCCACAA-3′. The PCR products were analyzed by electrophoresis in 1.5% agarose gels. The intensity of each band was photographed and quantified by using a Bio-Rad iQ5 system (Bio-Rad Laboratories, CA, USA) as a ratio of a target gene over GAPDH.

### 2.5. Western Blot Analysis

Frozen LV specimens were dispersed mechanically in the lysis buffer. The lysate was centrifuged at 10,000 r/min for 10 min at 4°C, and the supernatant was collected. The protein concentrations were quantified using an enhanced BCA Protein Assay Kit (Beyotime Biotechnology, Haimen, China). After the protein concentrations were determined, 30-50 *μ*g of protein was separated by SDS-PAGE and electrophoretically transferred onto PVDF membrane and blocked overnight with 1% skim milk in TBS at 4°C. The blots were incubated with specific primary antibodies against Bcl-2, Bax, Akt, and p-Akt for 2 h at room temperature, washed, and then incubated with appropriate peroxidase-conjugated secondary antibodies. Bcl-2, Bax, Akt, and phosphor-Akt antibodies and goat anti-rabbit IgG conjugated with peroxidase were obtained from Santa Cruz Biotechnology, Santa Cruz, CA, USA, and Cell Signaling technology, Inc., USA, respectively. The immune complexes were visualized by using ECL detection reagents following the manufacturer's protocol. Densitometric analysis was carried out with a Western blotting detection system (Alpha Innotech, USA). The band intensity for each sample was analyzed, and protein expression was normalized to GAPDH.

### 2.6. Statistical Analysis

The data are presented as mean ± SD. Statistical analysis was performed using SPSS version 15.0. The differences between groups were determined using one-way ANOVA for repeated measures. A value of *P* < 0.05 was considered statistically significant.

## 3. Results

### 3.1. (*Z*)-2-Acetoxy-3-(3,4-Dihydroxyphenyl) Acrylic Acid Improves Cardiac Function

The synthetic route of (*Z*)-2-acetoxy-3-(3,4-dihydroxyphenyl) acrylic acid was performed as described in detail previously in our laboratory [18].

Table 1 lists the effects of (*Z*)-2-acetoxy-3-(3,4-dihydroxyphenyl) acrylic acid on rat physiological and hemodynamic parameters 6 weeks after MI. Compared with the sham operation group, the MI group presented significantly increased LV end-diastolic Pressure (LVEDP), in addition, decreased Left ventricular systolic Pressure(LVSP) and maximum rate of *dP*/*dt*. In the MI group, *dP*/*dt* as an index of myocardial contractility was significantly reduced compared with the sham operation group. In addition, in the 15 mg/kg (*Z*)-2-acetoxy-3-(3,4-dihydroxyphenyl) acrylic acid-treated group, a significant improvement of *dP*/*dt* was observed (*P* < 0.05). These data indicated that (*Z*)-2-acetoxy-3-(3,4-dihydroxyphenyl) acrylic acid promotes cardiac function at a lower concentration.

### 3.2. Dose-Dependent (*Z*)-2-Acetoxy-3-(3,4-Dihydroxyphenyl) Acrylic Acid Protection against Oxidative Stress in Failing Hearts

**Figure 1:**
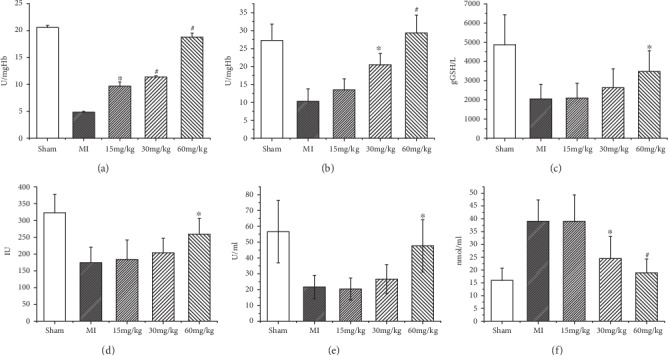
The antioxidant effects of (*Z*)-2-acetoxy-3-(3,4-dihydroxyphenyl) acrylic acid were implicated by the enhanced activities of catalase (CAT) (a), superoxide dismutase (SOD) (b), glutathione (GSH) (c), glutathione peroxidase (GSH-Px) (d), and GST and the levels of glutathione S-transferase (GST) (e) in the serum. On the other hand, (*Z*)-2-acetoxy-3-(3,4-dihydroxyphenyl) acrylic acid treatment reduced levels of serum malondialdehyde (MDA) (f) in a dose-dependent manner. ^#^*P* < 0.05 versus sham, ^∗^*P* < 0.05 versus MI, and *n* = 6 in each group.

The antioxidant effects of (*Z*)-2-acetoxy-3-(3,4-dihydroxyphenyl) acrylic acid were implicated by the enhanced activities of CAT, SOD, and GSH-Px and the levels of GSH in the serum, as well as the protein levels of CAT, SOD-1, and GPx in the left ventricle (Figures 1(a)–1(e)). On the other hand, (*Z*)-2-acetoxy-3-(3,4-dihydroxyphenyl) acrylic acid treatment reduced the levels of serum MDA in a dose-dependent manner (Figure 1(f)). In addition, (*Z*)-2-acetoxy-3-(3,4-dihydroxyphenyl) acrylic acid also increased the total antioxidant capacities and in parallel decreased the lipid peroxidation levels in the serum and left ventricle in a dose-dependent manner. The GSH content in the (*Z*)-2-acetoxy-3-(3,4-dihydroxyphenyl) acrylic acid treatment (15, 30, and 60 mg/kg) group was lower than that in the control group (*P* < 0.05). After treatment with three different concentrations of (*Z*)-2-acetoxy-3-(3,4-dihydroxyphenyl) acrylic acid treatment, GSH levels were higher in the treatment group than in the MI group (*P* < 0.05). The higher the concentration of (*Z*)-2-acetoxy-3-(3,4-dihydroxyphenyl) acrylic acid (15, 30, and 60 mg/kg), the higher the GSH concentration was. In addition, the levels of GSH-Px and GST were higher in the treatment group than in the MI group. Third, the levels of many additional enzymes, including CAT, MDA, and SOD, were also significantly different in the treatment group compared with the MI group. The CAT levels in the treatment group were significantly higher than those in the model group (*P* < 0.05). Similarly, the SOD levels were similar to the CAT levels. Lastly, the MDA concentration in the treatment group of HF rats was lower than that detected in the MI group. The posttreatment values for GSH, GSH-Px, CAT, SOD, and MDA all implied that (*Z*)-2-acetoxy-3-(3,4-dihydroxyphenyl) acrylic acid had the ability to protect antioxidant effects from a failing heart.

### 3.3. (*Z*)-2-Acetoxy-3-(3,4-Dihydroxyphenyl) Acrylic Acid Attenuates Inflammatory Cytokine Expression in Failing Hearts

**Figure 2:**
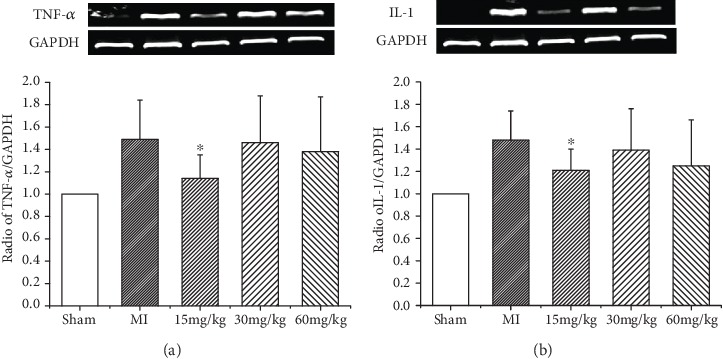
Expression of TNF-*α* and IL-1*β* mRNA. (*Z*)-2-Acetoxy-3-(3,4-dihydroxyphenyl) acrylic acid treatment (15, 30, and 60 mg/kg) reduces inflammatory cytokine expression in failing hearts. (a) TNF-*α* mRNA level. (b) IL-1*β* mRNA level. Values are expressed as mean ± SD, ^∗^*P* < 0.05 versus MI. *n* = 6 in each group.

The protective effect of (*Z*)-2-acetoxy-3-(3,4-dihydroxyphenyl) acrylic acid was evaluated by analyzing the expression levels of proinflammatory genes in the peri-infarct area using RT-PCR. In the MI group, an upregulation in TNF-*α* mRNA levels was observed compared with the sham group. This effect was partially reversed by (*Z*)-2-acetoxy-3-(3,4-dihydroxyphenyl) acrylic acid treatment, especially at the 15 mg/kg dose (Figure 2(a)). Similarly, a significant increase in IL-1*β* mRNA expression was detected in the MI group, which was attenuated by treatment with (*Z*)-2-acetoxy-3-(3,4-dihydroxyphenyl) acrylic acid at a 15 mg/kg dose (Figure 2(b)). These data suggested that (*Z*)-2-acetoxy-3-(3,4-dihydroxyphenyl) acrylic acid is a potent inhibitor of the inflammatory process.

### 3.4. (*Z*)-2-Acetoxy-3-(3,4-Dihydroxyphenyl) Acrylic Acid Reduces Cardiomyocyte Apoptosis and Increases Akt Phosphorylation in Failing Hearts

**Figure 3:**
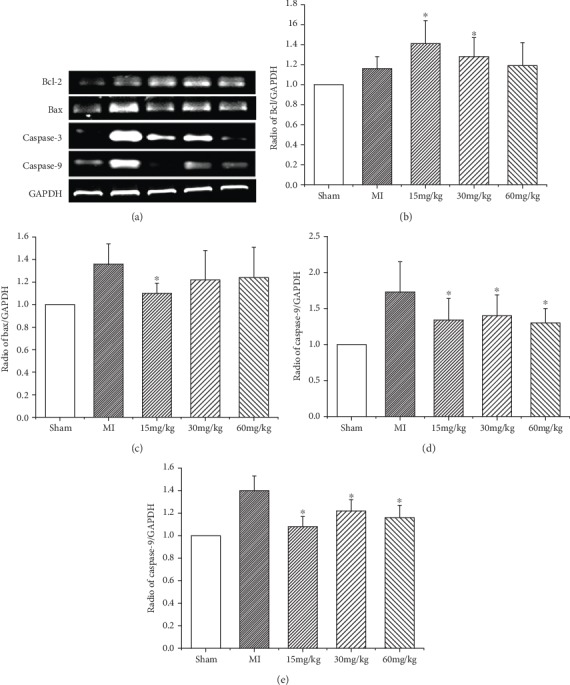
(a) Expression of Bcl-2, Bax, caspase-3, and caspase-9 mRNA. (*Z*)-2-Acetoxy-3-(3,4-dihydroxyphenyl) acrylic acid treatment (15, 30, and 60 mg/kg) reduces the mRNA expression levels of apoptosis-related molecules in heart tissue. (b–e) Relative Bcl-2, Bax, caspase-3, and caspase-9 mRNA levels. Values are expressed as mean ± SD, ^∗^*P* < 0.05 versus MI. *n* = 6 in each group.

We next determined the inhibitory effects of (*Z*)-2-acetoxy-3-(3,4-dihydroxyphenyl) acrylic acid on cardiomyocyte apoptosis. We measured the protein and mRNA expression levels of several apoptosis-related molecules in the peri-infarct area. RT-PCR revealed that the expression level of Bcl-2 was increased in the MI group and the (*Z*)-2-acetoxy-3-(3,4-dihydroxyphenyl) acrylic acid-treated group compared with the sham operation group. (*Z*)-2-Acetoxy-3-(3,4-dihydroxyphenyl) acrylic acid treatment resulted in a significant increase in Bcl-2 mRNA in the treatment group compared with the MI group (Figure 3(a)). In contrast, the mRNA levels of the proapoptotic molecules Bax, caspase-3, and caspase-9 were significantly reduced in the (*Z*)-2-acetoxy-3-(3,4-dihydroxyphenyl) acrylic acid-treated group compared with the MI group (Figure 3(b)). Western blot analysis confirmed the RT-PCR results. Indeed, a significant increase in Bcl-2 protein levels and a reduction in Bax protein levels were detected in the (*Z*)-2-acetoxy-3-(3,4-dihydroxyphenyl) acrylic acid-treated group compared with the MI group (Figures 3(a) and 3(b)). Moreover, these changes appeared to be associated with an increase in the phosphorylation of Akt in the MI and (*Z*)-2-acetoxy-3-(3,4-dihydroxyphenyl) acrylic acid-treated animals.

**Figure 4:**
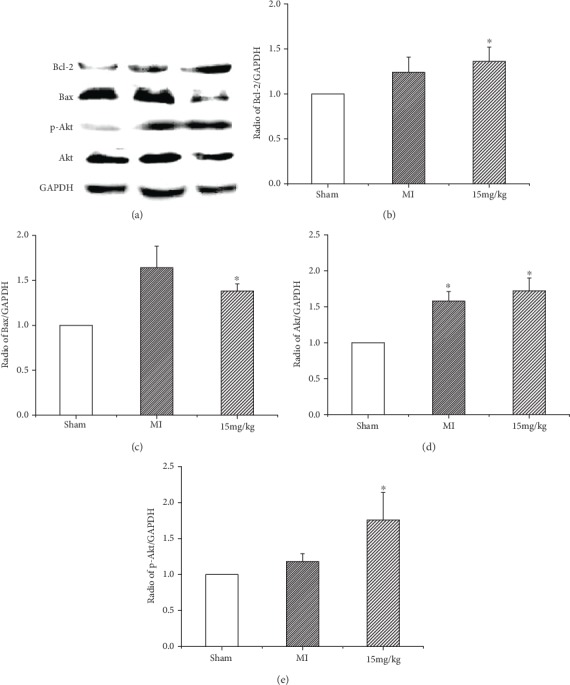
(a) Expression of Bcl-2, Bax, Akt, and p-Akt protein. (*Z*)-2-Acetoxy-3-(3,4-dihydroxyphenyl) acrylic acid treatment reduces the protein expression levels of apoptosis-related molecules in failing hearts. (b–e) Representative Western blots of Bcl-2, Bax, Akt, and p-Akt protein levels. Values are expressed as mean ± SD, ^∗^*P* < 0.05 versus MI. *n* = 6 in each group.

Western blot analysis revealed that Akt was activated in the hearts of MI animals after 6 weeks. In the (*Z*)-2-acetoxy-3-(3,4-dihydroxyphenyl) acrylic acid-treated group, a significant increase in the phosphorylation of Akt was observed compared with the MI group (Figure 4). The combined findings of this study indicate that the activation of Akt mediated the expression of apoptosis-related molecules.

### 3.5. (*Z*)-2-Acetoxy-3-(3,4-Dihydroxyphenyl) Acrylic Acid Reduces Adverse Ventricular Remodeling

**Figure 5:**
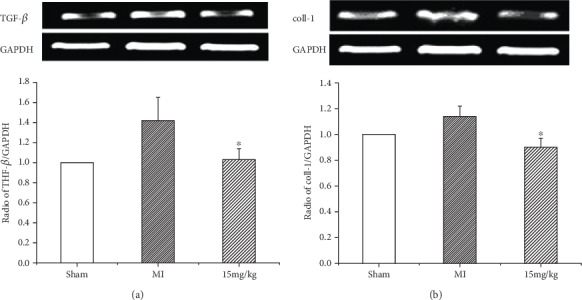
(a) Expression of TGF-*β* mRNA. (*Z*)-2-Acetoxy-3-(3,4-dihydroxyphenyl) acrylic acid reduces adverse ventricular remodeling in failing hearts. (b) Expression of collagen type I mRNA. (*Z*)-2-Acetoxy-3-(3,4-dihydroxyphenyl) acrylic acid reduces adverse ventricular remodeling in failing hearts. Values are expressed as mean ± SD, ^∗^*P* < 0.05 versus MI. *n* = 6 in each group.

Six weeks after MI, collagen deposition was detected by measuring the expression levels of TGF-*β* and collagen type I mRNA in the noninfarct area. RT-PCR revealed that TGF-*β* and collagen type I mRNA were significantly upregulated in the MI group. (*Z*)-2-Acetoxy-3-(3,4-dihydroxyphenyl) acrylic acid treatment could reduce the expression of these two molecules, although the differences were not statistically significant (Figure 5).

## 4. Discussions

Our data indicated that treatment with (*Z*)-2-acetoxy-3-(3,4-dihydroxyphenyl) acrylic acid improved cardiac function, particularly by increasing *dP*/*dt*, an important marker of left ventricular function in chronically failing hearts. Among the doses of 15, 30, and 60 mg/kg, using the lowest dose (15 mg/kg) provided the best protective effects. Although doses lower than 15 mg/kg were not used, the present results suggested that treatment with (*Z*)-2-acetoxy-3-(3,4-dihydroxyphenyl) acrylic acid at lower doses may provide better benefits. Thus, the optimal dose remains to be determined.

The pathological mechanism of myocardial infarction is multifactorial. Previous studies have shown that a large number of reactive oxygen species are generated; studies also have confirmed that the production of a large number of oxygen free radicals after ischemia is one of the main mechanisms of cardiomyocyte injury. Our results show that (*Z*)-2-acetoxy-3-(3,4-dihydroxyphenyl) acrylic acid can reduce the leakage rate of LDH, reduce the production of MDA, inhibit the expression of apoptosis-related factors, and show good myocardial protection. Those endogenous antioxidant enzymes, SOD and catalase, can detoxify reactive oxygen species and thus rescue cells from oxidative damage. The protective effects of (*Z*)-2-acetoxy-3-(3,4-dihydroxyphenyl) acrylic acid appear to be associated with the attenuation of inflammatory cytokine expression and the inhibition of myocardial apoptosis. Expression of the TNF-*α* and IL-1*β* genes was significantly reduced. It is known that inflammatory responses and cytokine release play an active role in heart damage following myocardial infarction [20, 21]. A large number of reports have demonstrated that the expression of proinflammatory cytokines is directly related to the degree of heart failure and inversely related to survival [20, 22]. Indeed, results obtained from several animal studies and some clinical trials suggest that suppression of inflammatory cytokines may improve cardiac performance [23]. Interestingly, studies from Kurrelmeyer et al. demonstrated that transgenic mice with knocked-out TNF receptors showed an increase in apoptosis induced by acute coronary occlusion, suggesting a protective TNF-*α* effect in the myocardium [24]. However, the protective role of TNF-*α* in heart failure models remains unclear. In our study, treatment with (*Z*)-2-acetoxy-3-(3,4-dihydroxyphenyl) acrylic acid significantly improved *dP*/*dt*, which was related to the reduction of the gene expression levels of TNF-*α* and IL-1*β*. It is conceivable that (*Z*)-2-acetoxy-3-(3,4-dihydroxyphenyl) acrylic acid plays a beneficial role in cardiac repair after MI by significantly reducing the inflammatory response in the infracted myocardium.

In addition to the potent anti-inflammatory effects, the protective effects of (*Z*)-2-acetoxy-3-(3,4-dihydroxyphenyl) acrylic acid were supplemented by inhibiting cell apoptosis via the promotion of Akt phosphorylation and modulation of the gene expression of apoptosis-related molecules. Cytotoxic cytokines and tissue-damaging agents are two major inducers of apoptosis. Cytotoxic cytokines induce apoptosis by binding to their receptors. Activation of these receptors causes activation of caspase-8, which can then activate other caspases as well as the proapoptotic members of the Bcl-2 family. The activated caspase-8 leads to the release of cytochrome c from the mitochondria to the cytoplasm, which activates caspase-3, leading to inevitable cell death [25]. Activation of caspase-3 has been reported in the heart tissues of a number of species during end-stage heart failure, including humans, sheep, and rabbits [26]. Several studies have demonstrated the feasibility of suppressing caspases to prevent heart failure. IDN-1965, a small molecule nonpeptide caspase inhibitor, inhibits a broad spectrum of caspases and has been shown to be effective in inhibiting apoptosis and liver injury induced by cytokines, suggesting a new basis for developing pharmaceutical agents against heart failure [27]. In our study, treatment with (*Z*)-2-acetoxy-3-(3,4-dihydroxyphenyl) acrylic acid reduced the mRNA expression levels of caspase-3 and caspase-9, indicating (*Z*)-2-acetoxy-3-(3,4-dihydroxyphenyl) acrylic acid may be a potent caspase inhibitor.

It is well known that an imbalance of proapoptotic versus prosurvival members in the Bcl-2 family plays an important role in apoptosis of various cell types [28]. The prosurvival factors Bcl-xL and Bcl-2 have been observed in patients with end-stage heart failure and in cultured cardiomyocytes upon exposure to cytotoxic cytokines [29–31]. The proapoptotic factor Bax has also been found in cultured ventricles in a chronic pressure-overloaded rabbits' heart model [32]. Although Bax gene expression was not found to be altered in failing human hearts, there is much evidence supporting the role of the Bcl-2 family in apoptosis. For example, the overexpression of Bcl-2 or Bcl-xL prevents mitochondrial membrane permeability transition and Bax-mediated release of cytochrome c [33]. Our study demonstrated that Bcl-2 and Bax gene expression increased in the MI group six weeks after surgery. (*Z*)-2-Acetoxy-3-(3,4-dihydroxyphenyl) acrylic acid treatment significantly decreased Bax gene expression and further improved mRNA expression of Bcl-2, which is associated with improved heart function. Akt is a key modulator in the apoptosis survival pathway. Increased Akt/GSK-3*β* phosphorylation plays an important role in promoting cell survival. Akt inactivates GSK-3*β* and then blocks cytochrome c release and caspase-3 activation. Our results indicated that (*Z*)-2-acetoxy-3-(3,4-dihydroxyphenyl) acrylic acid increased phosphorylated Akt levels compared to the untreated animals in the MI group, further indicating that the protective effect of (*Z*)-2-acetoxy-3-(3,4-dihydroxyphenyl) acrylic acid was achieved by inhibiting myocardial cell apoptosis.

Ventricular remodeling is another characteristic of heart failure. Many studies have shown that failure to prevent cardiomyocyte apoptosis and suppression of proinflammatory cytokines are involved in mediating cardiomyocyte hypertrophy and ventricular remodeling. Our study indicated that treatment with (*Z*)-2-acetoxy-3-(3,4-dihydroxyphenyl) acrylic acid decreased TGF-*β* and collagen type I gene expression, which was not the result that we expected. This phenomenon may be associated with differences between animal species of animals or sample amount. Our next study will further confirm the effects of (*Z*)-2-acetoxy-3-(3,4-dihydroxyphenyl) acrylic acid on other animal models, such as murine, rabbit, or porcine models.

## 5. Conclusions

**Figure 6:**
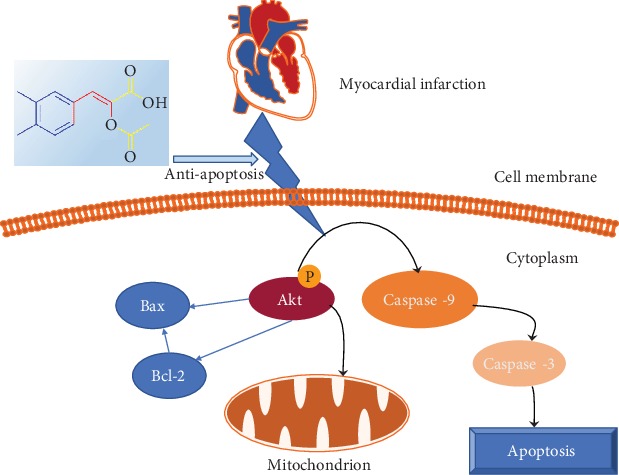
Potential mechanisms involved in the cardioprotective effect of (*Z*)-2-acetoxy-3-(3,4-dihydroxyphenyl) acrylic acid by inhibition of apoptosis in cardiomyocytes.

The present study demonstrates that (*Z*)-2-acetoxy-3-(3,4-dihydroxyphenyl) acrylic acid treatment was useful in treating post-MI heart failure by preferentially modulating the local inflammatory response and inhibiting myocardial apoptosis as evidenced by decreasing TNF-*α*, IL-1*β*, caspase-3, and capase-9 expression levels. In addition, the treatment improved outcomes by modulating the Akt prosurvival pathway. Taken together, these results suggest that (*Z*)-2-acetoxy-3-(3,4-dihydroxyphenyl) acrylic acid may be a potent chemical agent that can be used to protect failing hearts (Figure 6).

## Figures and Tables

**Table 1:** Cardiac parameter changes following (*Z*)-2-acetoxy-3-(3,4-dihydroxyphenyl) acrylic acid injection at 15, 30, and 60 mg/kg concentrations six weeks after MI. Data are expressed as mean ± SD. ^#^*P* < 0.05 versus sham, ^∗^*P* < 0.05 versus MI, and *n* = 6 in each group.

Variable	Sham	MI	MI+acrylic acid (15)	MI+acrylic acid (30)	MI+acrylic acid (60)
Body weight (g)	396 ± 62.12	310 ± 38.72	343 ± 41.43	354 ± 27.33	352 ± 18.18
Heart weight (mg)	940 ± 67	936 ± 164	963 ± 111	923 ± 90	896 ± 110
Heart/body weight (mg/g)	2.6 ± 0.1	3.1 ± 0.2	2.7 ± 0.3	2.6 ± 0.1^∗^	2.7 ± 0.1
Left ventricular systolic pressure (LVSP) (mmHg)	112 ± 32	101 ± 5^#^	107 ± 8	105 ± 10	101 ± 13
LV end-diastolic pressure (LVEDP) (mmHg)	2.9 ± 0.67	13.2 ± 7.5^#^	9.65 ± 1.6	8.2 ± 2.0	13.1 ± 4.8
+*dP*/*dt* max (mmHg)	5649 ± 1860	2564 ± 131^#^	3407 ± 239^∗^	3083 ± 92	3232 ± 378

## Data Availability

The Cardiac parameter changes data, enhanced activities of catalase (CAT), superoxide dismutase (SOD), glutathione peroxidase (GSH-PX) and the levels of glutathione (GSH) in the serum, the protein and mRNA levels of signals in the left ventricle used to support the findings of this study are available from the corresponding author upon request.
